# Advanced brain aging: relationship with epidemiologic and genetic risk factors, and overlap with Alzheimer disease atrophy patterns

**DOI:** 10.1038/tp.2016.39

**Published:** 2016-04-05

**Authors:** M Habes, D Janowitz, G Erus, J B Toledo, S M Resnick, J Doshi, S Van der Auwera, K Wittfeld, K Hegenscheid, N Hosten, R Biffar, G Homuth, H Völzke, H J Grabe, W Hoffmann, C Davatzikos

**Affiliations:** 1 Institute for Community Medicine, University of Greifswald, Greifswald, Germany; 2 Department of Radiology, Center for Biomedical Image Computing and Analytics, University of Pennsylvania, Philadelphia, PA, USA; 3 Department of Psychiatry, University of Greifswald, Greifswald, Germany; 4 Department of Pathology & Laboratory Medicine, Institute on Aging, Center for Neurodegenerative Disease Research, University of Pennsylvania, Philadelphia, PA, USA; 5 Laboratory of Behavioral Neuroscience, Biomedical Research Center, National Institute on Aging, Baltimore, MD, USA; 6 German Center for Neurodegenerative Diseases (DZNE), Rostock/Greifswald, Greifswald, Germany; 7 Department of Radiology, University of Greifswald, Greifswald, Germany; 8 Department of Dentistry, University of Greifswald, Greifswald, Germany; 9 Institute for Genetics and Functional Genomics, University of Greifswald, Greifswald, Germany

## Abstract

We systematically compared structural imaging patterns of advanced brain aging (ABA) in the general-population, herein defined as significant deviation from typical BA to those found in Alzheimer disease (AD). The hypothesis that ABA would show different patterns of structural change compared with those found in AD was tested via advanced pattern analysis methods. In particular, magnetic resonance images of 2705 participants from the Study of Health in Pomerania (aged 20–90 years) were analyzed using an index that captures aging atrophy patterns (Spatial Pattern of Atrophy for Recognition of BA (SPARE-BA)), and an index previously shown to capture atrophy patterns found in clinical AD (Spatial Patterns of Abnormality for Recognition of Early Alzheimer's Disease (SPARE-AD)). We studied the association between these indices and risk factors, including an AD polygenic risk score. Finally, we compared the ABA-associated atrophy with typical AD-like patterns. We observed that SPARE-BA had significant association with: smoking (*P<*0.05), anti-hypertensive (*P<*0.05), anti-diabetic drug use (men *P<*0.05, women *P*=0.06) and waist circumference for the male cohort (*P<*0.05), after adjusting for age. Subjects with ABA had spatially extensive gray matter loss in the frontal, parietal and temporal lobes (false-discovery-rate-corrected *q*<0.001). ABA patterns of atrophy were partially overlapping with, but notably deviating from those typically found in AD. Subjects with ABA had higher SPARE-AD values; largely due to the partial spatial overlap of associated patterns in temporal regions. The AD polygenic risk score was significantly associated with SPARE-AD but not with SPARE-BA. Our findings suggest that ABA is likely characterized by pathophysiologic mechanisms that are distinct from, or only partially overlapping with those of AD.

## Introduction

Aging has been associated with cognitive impairment affecting working memory, processing speed, executive function and episodic memory,^1^ but different mechanisms and underlying brain changes have been related to each cognitive domain. Cognitive functions associated with frontal cortex structures and networks, particularly processing speed and working memory, have been associated with ‘normal' brain aging (BA)^1, 2^ and vascular-related white matter changes. Episodic memory impairment in turn has been attributed to Alzheimer disease (AD), the prevalence of which exponentially increases with age. AD is characterized by tau pathology spreading from the medial temporal lobe and neocortical widespread amyloid beta deposition. Amyloid- and tau-independent mechanisms like mitochondrial dysfunction and oxidative stress have been linked to BA,^3^ although this does not exclude that the same aging-related mechanisms can lead to increased AD-related pathology.

Findings from previous studies have shown that BA in individuals without concurrent pathology is associated with pronounced gray matter loss, particularly in frontal and parietal lobes,^4, 5^ whereas amnestic mild cognitive impairment and AD subjects have shown atrophy patterns in the temporal lobe, hippocampus and parahippocampal gyrus.^6^ In addition, co-morbid conditions such as type 2 diabetes mellitus, hypertension and arteriolosclerosis are also associated with brain atrophy^7, 8, 9, 10, 11, 12^ and might have an additive effect on atrophy related to BA. While many studies independently showed spatially specific atrophy patterns occurring with normal aging or due to disease, structural brain changes in advanced BA (ABA), defined as significant deviation from typical BA trajectories, have not been systematically compared with AD-like brain changes in population-based studies. In addition, whether different co-morbid and genetic conditions are associated with BA and AD is still uncovered in the general community.

We hypothesized that ABA will show a pattern of brain atrophy that is distinct and only partially overlapping to the one that has been described for AD. To assess ABA and AD-like patterns a traditional approach using simple radiological measures like hippocampal volume,^13^ which is commonly used to investigate brain changes related to aging and AD, might not be able to capture the complex spectrum of changes, and more sophisticated methods are required. Herein, we leverage advanced pattern analysis techniques^14, 15, 16, 17, 18^ to derive a new quantitative index for brain changes as a function of age (Spatial Pattern of Atrophy for Recognition of BA (SPARE-BA)), and to compare those with spatial brain atrophy patterns specifically found in clinically diagnosed AD cases, using the Spatial Patterns of Abnormality for Recognition of Early Alzheimer's Disease (SPARE-AD) index^14, 18^ in a large sample from the population-based Study of Health in Pomerania (SHIP) that spanned a wide age range (20–90 years, *n*=2705).^19^

To our knowledge, the current study is the first to employ high-dimensional pattern recognition techniques to assess ABA patterns and to determine similarities and differences with clinical AD patterns in a large population-based cohort spanning almost the entire adulthood age range.

## Materials and methods

### Participants of SHIP

We included in this study participants form the population-based SHIP, which is lead by the Institute for Community Medicine at the University Medicine Greifswald. SHIP included in the latest follow-up whole-body magnetic resonance images (MRI)^19, 20, 21^ Its neuroimaging component has followed 3256 individuals (aged 20–90 years). Expert radiologists have inspected brain MRI scans for artifacts and clinical findings. We excluded scans based on existence of following clinical criteria (*n*=150): clinical stroke, multiple sclerosis, epilepsy, cerebral tumor, intracranial cyst or hydrocephalus, and high level of motion artifacts (*n*=98). Clinical data were not completely available for (*n*=182) subjects. Further exclusion occurred after quality control of the skull-stripping (*n*=121). The final sample was composed of 2705 subjects (full description is given in the Supplementary Table 1). The Ethics Committee of the University Medicine Greifswald approved SHIP.

### Data assessment in SHIP

Clinical data were collected by a computer-assisted face-to-face interview. Smoking was recorded in three categories, in particular: current smoking (⩾1 cigarette(s) per day), former smoking (⩾1 cigarette(s) per day) and never smoking. We subdivided the highest level of school education into three categories: <8, 8–10 and >10 years. For the assessment of leisure time physical activity (sportive exercise, for example, jogging) we specified the following groups: no activity, moderate (>0–1 h per week), high (1–2 h per week), as well as very high (>2 h per week) activity in summer and in winter.

Height and waist circumference were measured in cm. Blood pressure was recorded in mm Hg. Every medication was ascertained during the interview. We focused on anti-depressants, anti-hypertensives and anti-diabetic drugs. HbA1c, measured in %, was determined by high- performance liquid chromatography (Bio-Rad Diamat, Munich, Germany).

In our SHIP sample two cognitive tests were obtained: the Verbal Learning and Memory Test (VLMT, the German version for California VLMT^22^) for the sub-cohort SHIP-2 (*n*=772) and the Nurnberg Age Inventory (NAI)^23^ for the sub-cohort SHIP-Trend (*n*=1747).

### Image acquisition

We used T1-weighted MRI to measure regional patterns of aging, as well as Alzheimer's related brain atrophy. The image acquisition parameters of the whole-body MRI scans in SHIP have been described in the study by Hegenscheid *et al.*^20^ All images in this study were obtained using a 1.5 T scanner (Magnetom Avanto, Siemens Medical Systems, Erlangen, Germany) with an axial sequence and the following parameters: 1 × 1 mm in-plane spatial resolution, slice thickness=1.0 mm (flip angle 15°), echo time=3.4 ms and repetition time=1900 ms. The SHIP protocol included additional fluid-attenuated inversion recovery sequence, which we additionally used to mask out white matter hyperintesities.

### Image processing

Automated MRI analysis algorithms removed extra-cranial material (skull-stripping)^24^ (MASS V.1.0.0, http://www.cbica.upenn.edu/sbia/software/MASS/). MH performed quality control and all scans with low skull-stripping quality were excluded (*n*=121). Images were corrected for bias field^25^ and tissue segmented into gray matter, white matter, cerebrospinal fluid, as well as into a set of anatomical regions of interest.^26, 27, 28, 29^

We calculated regional volumetric maps, named RAVENS maps^30^ for gray matter, white matter and cerebrospinal fluid (DRAMMS V.1.41, http://www.cbica.upenn.edu/sbia/software/dramms/). The RAVENS approach has been extensively validated.^14, 30, 31, 32, 33^ We performed voxel-wise analysis of the gray matter RAVENS maps to inspect the spatial patterns of volumetric differences between groups (AFNI tools V.2008.07.18.1710, http://afni.nimh.nih.gov/afni/). White matter hyperintensities were segmented using a supervised-learning-based multi-modal segmentation algorithm^34^ applied on the T1 and the corresponding co-registered fluid-attenuated inversion recovery scans. The lesion segmentation results were then used to mask out the lesion area in the calculation of RAVENS maps and the tissue segmentations, so that they do not affect the derived atrophy measures by incorrectly assigning them to gray or white matter tissue types.

### MRI pattern classification

A high-dimensional pattern classification method was previously proposed to calculate SPARE-AD,^16, 33^ an index derived from the imaging data of cognitively normal older adults and clinical AD patients,^18^ to quantify atrophy patterns associated with AD. The pattern classifier was constructed to maximally differentiate between these two groups using a support vector machine.^35^ The SPARE-AD index has been shown to be predictive of conversion from normal cognition to mild cognition impairment^14^ and then to clinical AD^18^ with high accuracy.^14, 33^ We calculated the SPARE-AD values for the SHIP population using a model trained on the dataset defined in the study by Da *et al.*^18^

### Spatial Pattern of Atrophy for Recognition of BA

To derive an individualized index of age-related brain atrophy, we used two representative groups: a relatively younger group (⩽45 years, *n*=841, mean age±s.d. 36.7±6.3) and a relatively older group (⩾60 years, *n*=871, ages 68.2±5.6). A high-dimensional pattern classifier was constructed to maximally differentiate between these two groups using a support vector machine.^35^ The groups were well-balanced in terms of gender (53.6% and 54.3% women in the young and old groups, respectively). The model was then applied to all remaining subjects, yielding the SPARE-BA index for every individual; higher SPARE-BA values indicate lower brain atrophy (from the perspective of the spatial pattern associated with aging). SPARE-BA scores were also derived for the subjects of ages ⩽45 and ⩾60 using cross-validation (jack knifing), ensuring completely independent generalization.

### AD polygenic risk score

Janowitz *et al.*^36^ described Genotyping in SHIP. For the calculation of the AD polygenic risk score, we used 19 single nucleotide polymorphisms determined in recent study (17 008 cases and 37 154 controls).^37^ We calculated the AD polygenic risk score for all participants with the available genotyping (*n*=1837) as the sum of the number of alleles of every single nucleotide polymorphism weighted by the logarithm of the corresponding odds ratio^38^ This method for risk estimation was successfully applied in different studies^39, 40^ and recently for AD.^41^ Higher polygenic risk score is directly proportional to the development of AD. Supplementary Table 2 lists all single nucleotide polymorphisms included in the calculation of the polygenic risk score.

### Statistical analysis

We used ordinary least squares multivariable regression models to identify epidemiologic factors significantly associated with SPARE-BA in the whole SHIP sample (*n*=2705). Two models were built independently for male (*n*=1231) and female (*n*=1474) participants to assess gender-specific associations with BA atrophy patterns. To study the relationship between SPARE-AD and SPARE-BA, we focused on SHIP subjects ⩾65 years, since the prevalence of clinical AD starts to increase substantially after this age.^42^ To select subjects with ABA, we fit a linear regression between age and the SPARE-BA score, and calculated residual values of the SPARE-BA score after correcting for age. A *z*-score transformation is applied on the residuals. Subjects with *z*-score>0.5 were selected as the ‘resilient to aging' (RA) group and those with *z*-score<−0.5 as the ABA group. In Supplementary Analysis we defined clinical AD patterns between high and low SPARE-AD individuals determined in a similar way. Group differences were tested using independent Student's *t*-test. Analyses were performed using R software (V.3.1).^43^

## Results

### Prevalence of SPARE-BA in SHIP

**Figure 1:**
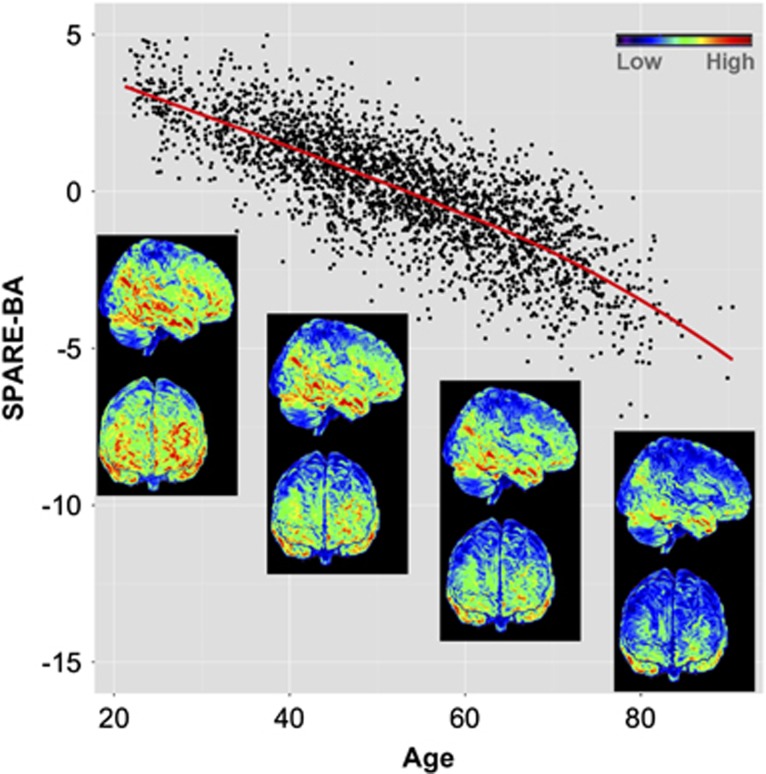
SPARE-BA values for all SHIP participants (*n*=2705) plotted against age. Red line represents local polynomial fitted curve. Higher/lower values indicate less/more aging-specific brain atrophy patterns, captured by the SPARE-BA index. Brain rendering shows the gray matter RAVENS (regional volumetric) maps for age groups 20–80. The color map represents lower RAVENS values (that is, less gray matter) in blue and higher RAVENS values (that is, more gray matter) in red. We observe a consistent decrease in gray matter volume in the cortex with age, which are particularly prominent in certain frontal, parietal and temporal brain regions. SPARE-BA, Spatial Pattern of Atrophy for Recognition of BA.

Figure 1 shows the SPARE-BA plotted as function of age for all participants (*n*=2705). There was a strong negative correlation between SPARE-BA and age with Pearson's correlation coefficient of *r*=−0.800 (*P<*0.0001). The Pearson's correlation coefficient between SPARE-BA and SPARE-AD was *r*=−0.491 in the whole sample and *r*=−0.515 in the sample with age ⩾65 years old (both with *P<*0.001).

**Figure 2:**
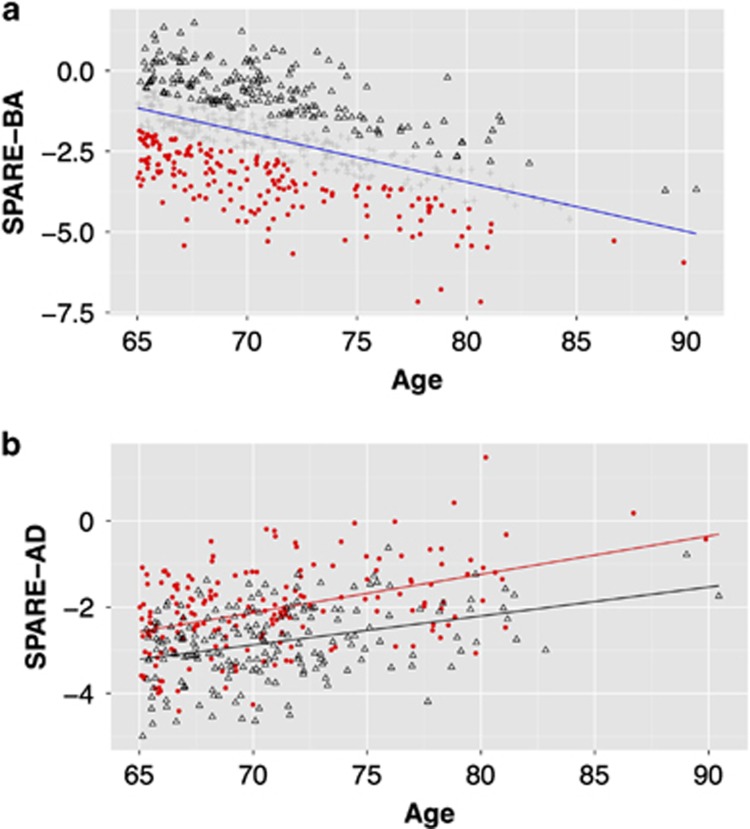
(**a**) SPARE-BA index for SHIP subjects ⩾65 years old. Values are reflecting relatively continuous progression of aging-related atrophy patterns with age. Based on SPARE-BA, we grouped these subjects into subjects with advanced brain aging (red dots; 0.5 s.d. below trend line) and resilient to brain aging (black triangles; 0.5 s.d. above trend line). (**b**) The relationship between age and SPARE-AD (reflecting clinical AD-like patterns of brain atrophy) in both groups. Advanced brain aging individuals have higher SPARE-AD values. SPARE-AD, Spatial Patterns of Abnormality for Recognition of Early Alzheimer's Disease; SPARE-BA, Spatial Pattern of Atrophy for Recognition of BA.

In subjects older than 65 years, we defined the ABA (*n*=179) and resilient to aging (RA, *n*=191) based on the SPARE-BA score being 0.5 s.d. below and above the regression line, respectively (Figure 2). The distributions of the SPARE-AD scores for the subjects in ABA and RA groups are shown in Figure 2. Mean SPARE-AD of the ABA group was higher than the mean SPARE-AD of the RA group (mean±s.d. was −2.051±0.987 and −2.813±0.842, respectively, independent Student's *t*-test: *P<*0.0001).

### Epidemiologic risk factors associated with SPARE-BA

Multivariable regression models on the whole sample revealed significant associations between lower SPARE-BA and older age. After discarding the effect of age, significant associations were found with smoking, sedentary life style, anti-hypertensive and anti-diabetic drugs in both genders (Table 1). Furthermore, greater waist circumference was also inversely associated with SPARE-BA in male subjects. In Supplementary Table 3 the associations of risk factors with SPARE-AD are reported.

### Spatial patterns of ABA atrophy in older adults (⩾65 years)

**Figure 3:**
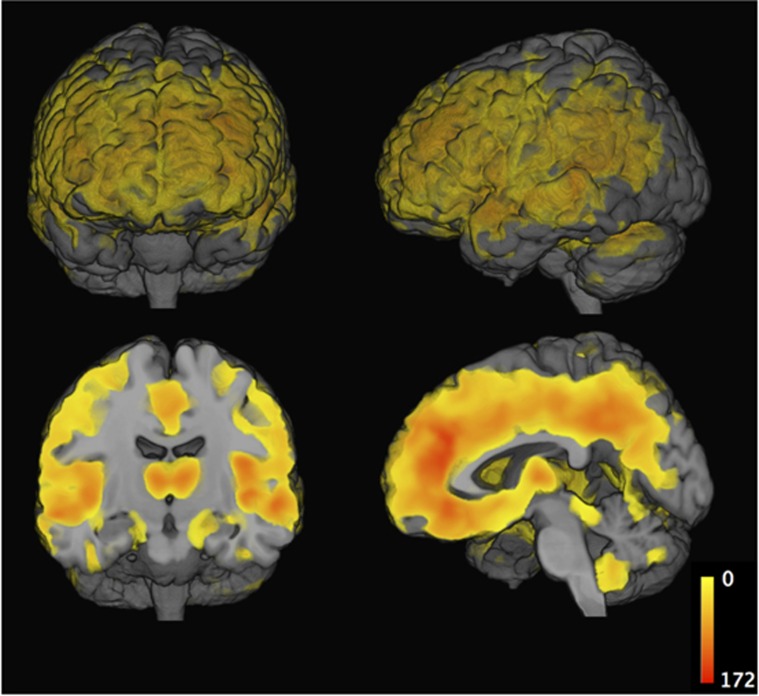
Regional gray matter volume differences between resilient aging (*n*=191) and advanced aging (*n*=179) subjects. Results are significant at level *P<*0.001, and all survived FDR correction with *q*<0.001. FDR, false discovery rate.

Figure 3 illustrates regions where ABA subjects showed lower gray matter volumes compared with RA subjects. Gray matter decrease was most significant in insular cortex, thalamus and cingulate cortex and further extended to frontal, inferior parietal and lateral temporal cortex. In a Supplementary Analysis, we computed regional atrophy patterns derived using continuous values of age-adjusted SPARE-BA scores, instead of using subjects dichotomized into ABA and RA groups. This analysis resulted in very similar regional patterns of atrophy, consistent with the ones shown in Figure 3 (Supplementary Figure 1).

**Figure 4:**
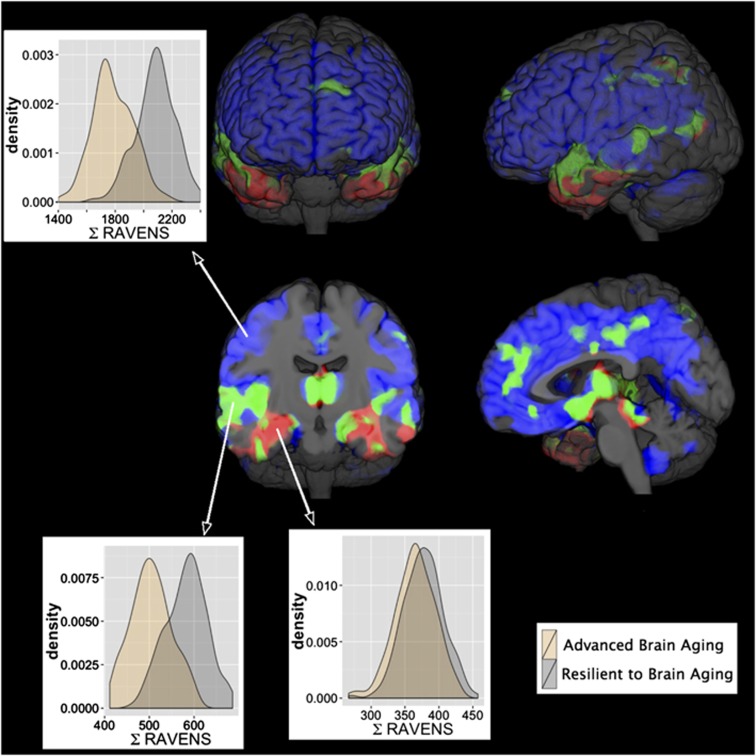
Patterns overlap. Blue: regions displaying significant regional atrophy patterns between resilient and ABA individuals; Red: regions displaying significantly AD-related patterns of atrophy in high vs low SPARE-AD individuals; Green: overlap of the blue and red regions (*P<*0.001 and FDR correction with *q*<0.001). Histograms are presented for gray matter volumes of blue/red/green regions for ABA and RA individuals. These results highlight the difference between advanced and clinical AD-like brain aging, and they also indicate that increased SPARE-AD values in advanced aging are entirely due to partial overlap (green) of underlying regions. ABA, advanced brain aging; FDR, false discovery rate; RA, resilient to aging; SPARE-AD, Spatial Patterns of Abnormality for Recognition of Early Alzheimer's Disease.

ABA showed a spatial pattern that deviated notably from previously reported spatial patterns of AD-related gray matter atrophy,^6, 14^ as well as from AD-related atrophy patterns we identified by comparing individuals in age-adjusted high and low SPARE-AD groups in the SHIP data (Supplementary Figure 2). Figure 4 illustrates the spatial patterns of regional volumetric differences between RA and ABA groups (in blue) and between age-adjusted low and high SPARE-AD groups (in red), as well as the overlap between both (in green). Most notable were the differences in the medial and inferior temporal lobe, including the hippocampal region that is more severely involved in AD as shown in Supplementary Figure 2.

While, within the blue and green regions the density distributions of RAVENS values show a clear shift between ABA and RA individuals—indicating significant volume reduction—the two groups did not have such a shift for the density distributions within the red region, indicating that the correlation between SPARE-BA and SPARE-AD is largely driven by the spatial overlap of respective patterns. We found similar results in terms of atrophy pattern distinctions after exclusion of possible impaired individuals according to their age-adjusted cognitive scores as sensitivity Supplementary Analysis (*n*=232 aged from 20 to 90, data not shown).

### AD polygenic risk score association with SPARE-BA

No significant association was found between SPARE-BA and the AD polygenic risk score for genotyped subjects neither in the whole age range sample (*n*=1689, *P*=0.676) nor in the subsample with age ⩾65 years (*n*=372, *P*=0.352). However, we found a close to significant association between SPARE-AD and the AD polygenic risk score in the whole age range sample (*P*=0.069) and significant association in the subsample with age ⩾65 years (*P*=0.016; Supplementary Results 1).

### Association with the available cognitive scores in SHIP

SPARE-BA was not significantly associated with the verbal learning memory score for SHIP-2 (*n*=730; *P*=0.340), and the NAI score for SHIP-Trend (*n*=1,637; *P*=0.410) after adjusting for age, gender and education in a linear regression model. Similarly, no significant association was found between SPARE-AD and cognitive scores (data not shown).

## Discussion

The relationship between ABA and AD-like structural brain changes has not been systematically studied in population-based studies. In a large neuroimaging cohort, we went beyond describing atrophy in predefined atlas based-regions, and we quantified atrophy patterns due to aging and AD using summary indices (SPARE-BA and SPARE-AD, respectively) derived from high-dimensional imaging data, leveraging advanced analytical techniques. We report that ABA individuals display patterns that are different from, albeit partially spatially overlapping with, patterns found in AD. This finding supports the hypothesis that distinct mechanisms might underlie lifetime BA and late-life neurodegeneration. However, by virtue of a partial overlap with AD-affected brain regions, ABA might be a co-morbidity leading to an earlier onset of dementia due to an additive effect of both mechanisms, as has been shown for coincident pathologies.^44, 45^

### BA patterns of atrophy and the associations with risk factors

ABA, defined as significant deviation from typical age-related BA trajectories in this population, was associated with significant gray matter volume reduction in widespread frontal and parietal regions and more restricted temporal lobe areas (Figure 3). Our results regarding frontal and parietal age-related atrophy are consistent with observations from Resnick *et al.,*^4^ who found that frontal and parietal lobes showed greater decline compared with temporal and lobar regions in cognitively normal aging individuals.^4^ Furthermore, we confirm observations of Raz *et al.*^46^that age is associated with differential shrinkage of frontal regions.

To expand the knowledge regarding BA and the mechanisms underlying the observed MRI patterns, we evaluated the impact of known vascular risk factors on SPARE-BA. Our findings of association between ABA and smoking are consistent with prior reports demonstrating that regional brain volume reductions are associated with smoking.^47^ We also observed that anti-hypertensive medication use was associated with ABA patterns. Anti-hypertensive medications could be considered a proxy for chronic hypertension, and thus, our findings are consistent with prior reports that hypertension is associated with brain atrophy,^11^ particularly in the frontal and temporal lobes.^48^ Results for waist circumference in men are in line with global loss and regional alterations in gray matter volume in obesity^49^ and obese men^50^ The gender-specific result might reflect different fat distribution patterns like the android fat distribution that is more relevant to brain alterations than gynoid fat distribution.^51^ Our observations regarding ABA patterns in diabetic men compared with men without diabetes are in line with a prior report indicating an association between brain atrophy and diabetes in smaller sample,^7, 52^ but other studies have also found associations between diabetes and brain atrophy in women.^53^ Our findings with respect to anti-depressants were only at the trend level and suggest a small effect given the large sample size. It is notable that anti-depressants are prescribed for patients suffering from pain, sleep disturbances and anxiety in addition to conditions associated with major depressive disorders. Effects of these other chronic diseases and their distress may lead to ABA. In addition, AD and other neurodegenerative diseases have been linked to psychiatric symptoms that can precede the dementia diagnosis.^54, 55^

These results support the hypothesis that ABA patterns are largely associated with several co-morbidities (android fat distribution, hypertension, diabetes, and perhaps, depression or chronic stress). While associations between most of these risk factors and brain atrophy have been reported previously, a contribution of our analysis lies in developing an age-specific index that increases the statistical power to detect BA-related changes, and showing that the studied factors may modify patterns of brain structure in a manner that might ‘accelerate' neurodegeneration related to the aging process. In addition, the large sample of this study enabled sex-stratified analyses of risk factors in relation to patterns of age-related atrophy. In addition, only few MR studies have such a statistical power, population-based sample and very high standardization protocol.

Multivariable regression models revealed risk factor associations with SPARE-AD that were different from those associated with SPARE-BA. In women, older age and smoking were associated with SPARE-AD. No significant risk factor other than age was associated with SPARE-AD in men, as reported in Supplementary Table 3. In the regression model for SPARE-AD, we found significant association when we included a quadratic age term in the model, indicating a non-linear relationship between age and greater atrophy in AD-related regions. This finding is in line with a previous report in the Baltimore Longitudinal Study of Aging (BLSA) cohort.^14^ Overall, the observed differences in risk factors associated with either SPARE-AD or SPARE-BA support the hypothesis that ABA is characterized by pathophysiologic mechanisms that are distinct from clinical AD-related atrophy patterns.

### Contribution of high-dimensional pattern classification techniques

An important contribution of our study is the use of advanced methods of high dimensional pattern classification for BA assessment, which allowed us to investigate in detail the spatial patterns of atrophy, and to derive individualized indices that were further correlated with epidemiologic and clinical factors. Our approach utilizes information from all brain regions jointly, thereby capturing the structural abnormality subtleties in BA, which is high dimensional in nature and goes beyond the small number of dimensions represented in one or few volumetric measures. Recently, Janowitz *et al.*^36^ analyzed prediction patterns for hippocampal volumes in SHIP. Interestingly the associated risk factors with hippocampal volume were similar to those associated with the aging patterns in the current study but different from the prediction patterns of SPARE-AD. This finding indicates that the hippocampus alone is unlikely to adequately reflect the complexity of neurodegeneration in AD, as previously demonstrated in the study by Fan *et al.*^15^

### Overlap between ABA and AD spatial patterns of atrophy

Figure 4 shows that the regions of the ABA-related spatial patterns of atrophy overlapped only partially with the clinical AD-related patterns. While ABA-like patterns were widespread in the brain (in blue), clinical AD-like patterns were spatially more localized (in red), mostly significant in several (especially medial) temporal lobe regions. The overlap between clinical AD-like and ABA-like regions (in green) existed mainly in parts of the hippocampus and in areas of the temporal lobe. These differences in the spatial distribution of atrophy patterns associated to ABA and to clinical AD suggest that ABA stems from distinct mechanisms, which potentially constitute a co-morbidity for clinical AD largely by virtue of affecting spatially overlapping brain regions.

### The AD polygenic risk score associations

Finally, the AD polygenic risk score was not significantly associated with SPARE-BA across the whole age range or in analyses restricted to older subjects, it was only close to significant association in the whole age range sample (*r*=0.044, *P*=0.069) and significantly associated with SPARE-AD in older subjects (*r*=0.124, *P*=0.016) as reported in Supplementary Analysis. This differences in association with polygenic risk score for SPARE-BA vs SPARE-AD offers additional support for the hypothesis that AD-related genetic risk leads to distinctive atrophy patterns. In fact the increase in prevalence of AD is more pronounced after age 65,^42^ which is in line with the significant association we found between SPARE-AD and the polygenic risk score at older age. The underlying disease process may start years before the AD diagnosis in elderly individuals. Singh-Manoux *et al.*^56^ showed that the brain function started to deteriorate as early as age 45. However, we did not find a significant association between AD polygenic risk score and SPARE-AD in the whole age range sample, likely reflecting the fact that only a small subset of individuals could be in a preclinical AD stage, or that SPARE-AD captures neurodegenerative changes occurring relatively later in the disease process.

This study has several strengths including the large sample size in a population-based sample and the use of novel pattern analysis approaches to investigate BA. However, this study has also limitations, which include the lack of longitudinal MRI scans and detailed clinical information for the complete SHIP cohort.

In summary, the current study is the first, to our knowledge, to employ high-dimensional pattern recognition techniques to assess BA patterns in a cohort of this size and show that it has a unique spatial pattern of brain atrophy that differs from the one found in AD.

## Figures and Tables

**Table 1:** Multivariable regression models for SPARE-BA in the whole SHIP sample included in this study (*n*=2705)

*Tables factor*	*Male* ⩾*20 years*, n=*1231*			*Female* ⩾*20 years,* n=*1474*		
	*Estimate*	*s.e.*	*P* value	*Estimate*	*s.e.*	*P* value
Age^2^, year^2^	0.000	0.000	0.012^a^	0.000	0.000	0.296
Age, year	−0.071	0.014	<0.0001^a^	−0.085	0.016	<0.0001^a^
Systolic blood pressure, mm Hg	0.002	0.002	0.323	−0.002	0.002	0.252
Glycated hemoglobin (HbA1c), %	−0.002	0.041	0.963	0.027	0.050	0.595
						
*Cigarette smoking*						
Ex−smoker	−0.169	0.070	0.015^a^	−0.156	0.069	0.023^a^
Current smoker	−0.325	0.082	<0.0001^a^	−0.144	0.081	0.075
						
Waist circumference, cm	−0.011	0.003	0.001^a^	−0.004	0.003	0.165
						
*Education*						
8–10 years	0.052	0.093	0.576	0.023	0.099	0.814
>10 years	−0.038	0.095	0.688	−0.059	0.107	0.585
						
*Physical activity*						
No sport-related activity	0.052	0.093	0.576	−0.035	0.085	0.680
>0–1 h per week	−0.038	0.095	0.688	0.104	0.099	0.295
>1–2 h per week	0.084	0.075	0.263	0.052	0.071	0.463
						
Anti-hypertensive drugs	−0.329	0.073	<0.0001^a^	−0.217	0.074	0.003^a^
Anti-diabetic drugs	−0.468	0.152	0.002^a^	−0.319	0.170	0.061
Anti-depressant drugs	−0.324	0.171	0.058	−0.207	0.111	0.061
	*R*^2^=0.731			*R*^2^=0.622		

Abbreviations: SHIP, Study of Health in Pomerania; SPARE-BA, Spatial Pattern of Atrophy for Recognition of Aging Brain.

a Significance at level *P<*0.05.
