# Supplementary material for: Advanced brain aging: relationship with epidemiologic and genetic risk factors, and overlap with Alzheimer disease atrophy patterns
Source: Transl Psychiatry. 2016 Apr 5;6(4):e775–. doi: 10.1038/tp.2016.39 (PMC4872397; doi:10.1038/tp.2016.39)
Supplement: Supplementary Information [file tp201639x1.doc]

**Supplemental Information**

Following data are to be reported as supplementary analysis in the SHIP cohort:

1. Supplementary Table 1: Description of the SHIP sample in this study
2. Supplementary Table 2: SNPs included in the calculation of the polygenic risk score
3. Supplementary Table 3: Multivariable regression models for Spatial Pattern of Abnormality for Recognition of Alzheimer’s Disease (SPARE-AD) in the whole SHIP sample
4. Supplementary Figure 1: Regions of significant gray matter atrophy computed using continuous values of age-adjusted SPARE-BA scores, for subjects older than 65 years old
5. Supplementary Figure 2: Regions of significant AD related gray matter atrophy between High and low SPARE-AD individuals for subjects older than 65
6. Supplementary Results 1: The association between SPARE-AD and the AD polygenic risk score

|  | Clinical prediction model | |  |
| --- | --- | --- | --- |
| Characteristic | Men  (n=1,231) | Women  (n=1,474) |  |
| Age, mean (SD), year | 52.5 (14.2) | 52.5 (13.1) |  |
| Body height, mean (SD), cm | 176.7 (6.7) | 163.8 (6.6)* |  |
| Education, N (%) |  |  |  |
| < 8 years | 198 (16.0) | 233 (15.8) |  |
| 8-10 years | 626 (50.8) | 878 (59.5) |  |
| > 10 years | 407 (33.0) | 363 (24.6) |  |
| Cigarette smoking, N (%) |  |  |  |
| Never-smoker | 390 (31.6) | 700 (47.4) |  |
| Ex-smoker | 552 (44.8) | 457 (31.0) |  |
| Current smoker | 289 (23.4) | 317 (21.5) |  |
| Physical activity, N (%) |  |  |  |
| No | 234 (19.0) | 262 (17.7) |  |
| >0 – 1 h/week | 132 (10.7) | 172 (11.6) |  |
| 1 - 2 h/week | 299 (24.2) | 459 (31.1) |  |
| >2 h/week | 566 (45.9) | 581 (39.4) |  |
| Waist circumference (cm), N (%) | 95.6 (10.8) | 83.9 (11.9)* |  |
| HbA1c (%) mean (SD) | 5.3 (0.8) | 5.2 (0.7) |  |
| Systolic blood pressure (mmHg), mean (SD) | 134.4 (15.8) | 121.9 (17.2)* |  |
| Medication |  |  |  |
| Antidepressant, N (%) | 38 (3.08) | 115 (7.8)* |  |
| Anti-diabetic, N (%) | 62 (5.0) | 56 (3.8) |  |
| Antihypertensive, N (%) | 403 (32.7) | 475 (32.2) |  |
|  |  |  |  |

*Significantly different at level P<.001

Supplementary Table 1 represents description of the SHIP sample included in the regression models

| **SNP** |
| --- |
| rs6656401 |
| rs6733839 |
| rs10948363 |
| rs11771145 |
| rs9331896 |
| rs983392 |
| rs10792832 |
| rs4147929 |
| rs9271192 |
| rs28834970 |
| rs11218343 |
| rs10498633 |
| rs35349669 |
| rs190982 |
| rs2718058 |
| rs1476679 |
| rs10838725 |
| rs17125944 |
| rs7274581 |
|  |

Supplementary Table 2 SNPs included in the calculation of the polygenic risk score

| Factor |  | | | | | | | | | | |
| --- | --- | --- | --- | --- | --- | --- | --- | --- | --- | --- | --- |
|  |  | Male ≥20 years  n=1,231 | | | |  | Female ≥20 years  n=1,474 | | | |  |
|  |  | Estimate | S.E. | P value |  | | Estimate | S.E. | | P value |  |
|  |  |  |  |  |  | |  |  | |  |  |
| **Age^2^, year^2^** |  | 0.001 | 0.000 | **<0.0001*** |  | | 0.001 | 0.000 | | **<0.0001*** |  |
|  |  |  |  |  |  | |  |  | |  |  |
| **Age, year** |  | -0.036 | 0.012 | **0.003*** |  | | -0.032 | 0.013 | | **0.011*** |  |
|  |  |  |  |  |  | |  |  | |  |  |
| **Systolic blood pressure, mm Hg** |  | 0.000 | 0.002 | 0.930 |  | | -0.001 | 0.002 | | 0.716 |  |
|  |  |  |  |  |  | |  |  | |  |  |
| **Glycated hemoglobin (HbA1c), %** |  | -0.056 | 0.034 | 0.099 |  | | -0.057 | 0.039 | | 0.140 |  |
|  |  |  |  |  |  | |  |  | |  |  |
| **Cigarette smoking** |  |  |  |  |  | |  |  | |  |  |
| Ex-smoker |  | 0.003 | 0.057 | 0.961 |  | | 0.107 | 0.053 | | **0.042*** |  |
| Current smoker |  | 0.042 | 0.067 | 0.537 |  | | 0.223 | 0.062 | | **0.001*** |  |
|  |  |  |  |  |  | |  |  | |  |  |
| **Waist circumference, cm** |  | 0.001 | 0.003 | 0.750 |  | | 0.001 | 0.002 | | 0.573 |  |
|  |  |  |  |  |  | |  |  | |  |  |
| **Education** |  |  |  |  |  | |  |  | |  |  |
| 8-10 years |  | 0.024 | 0.077 | 0.753 |  | | -0.101 | 0.076 | | 0.182 |  |
| >10 years |  | 0.002 | 0.078 | 0.976 |  | | -0.006 | 0.082 | | 0.946 |  |
|  |  |  |  |  |  | |  |  | |  |  |
| **Physical activity** |  |  |  |  |  | |  |  | |  |  |
| No sport related activity |  | 0.062 | 0.068 | 0.358 |  | | 0.030 | 0.065 | | 0.641 |  |
| >0-1 h/week |  | -0.018 | 0.083 | 0.824 |  | | -0.018 | 0.076 | | 0.813 |  |
| >1-2 h/week |  | 0.050 | 0.061 | 0.413 |  | | 0.053 | 0.054 | | 0.329 |  |
|  |  |  |  |  |  | |  |  | |  |  |
| **Antihypertensive drugs** |  | 0.052 | 0.060 | 0.387 |  | | -0.019 | 0.057 | | 0.739 |  |
|  |  |  |  |  |  | |  |  | |  |  |
| **Anti-diabetic drugs** |  | 0.093 | 0.125 | 0.458 |  | | -0.022 | 0.131 | | 0.869 |  |
|  |  |  |  |  |  | |  |  | |  |  |
| **Antidepressant drugs** |  | 0.159 | 0.140 | 0.256 |  | | 0.116 | 0.085 | | 0.172 |  |
|  |  |  |  |  |  | |  |  | |  |  |
|  |  | R^2^= 0.207 |  | |  | | R^2^= 0.155 | |  | |  |

* Significance at level p < 0.05, S.E: Standard Error

Supplementary Table 3: Multivariable regression models for Spatial Pattern of Abnormality for Recognition of Alzheimer’s Disease (SPARE-AD) in the whole SHIP sample included in this study (n=2705)


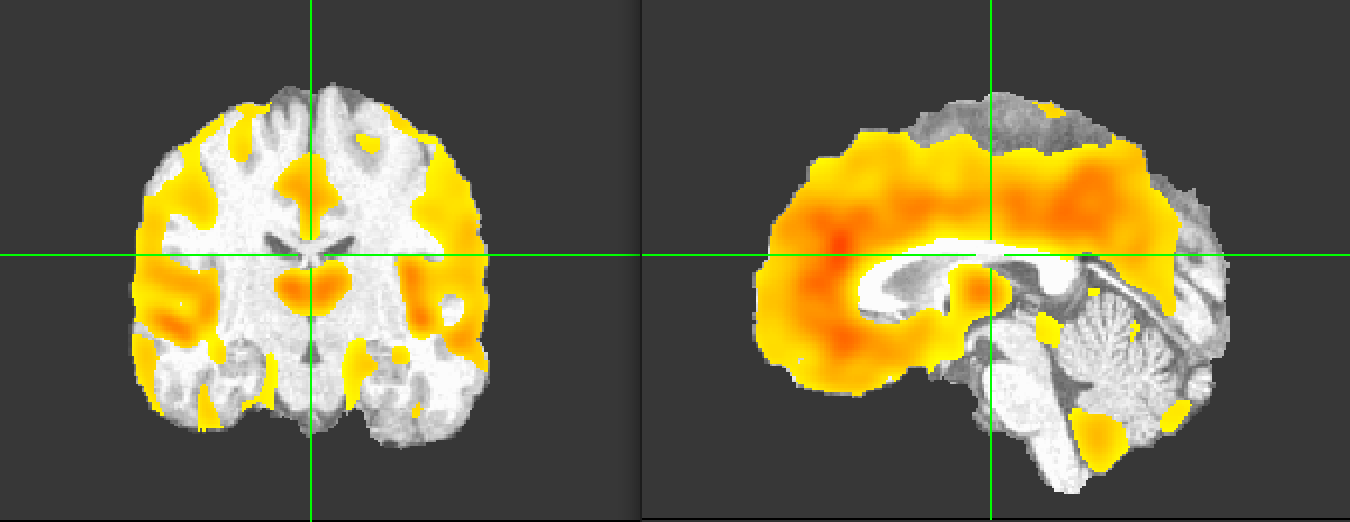


Supplementary Figure 1: Regions of gray matter atrophy that are significantly associated with age-adjusted SPARE-BA scores, for subjects older than 65 years old (left coronal view and right sagittal view). The color-map indicates lower (yellow) to higher (red) significance level. Voxels that survived FDR correction at significance level q<0.001 are shown.


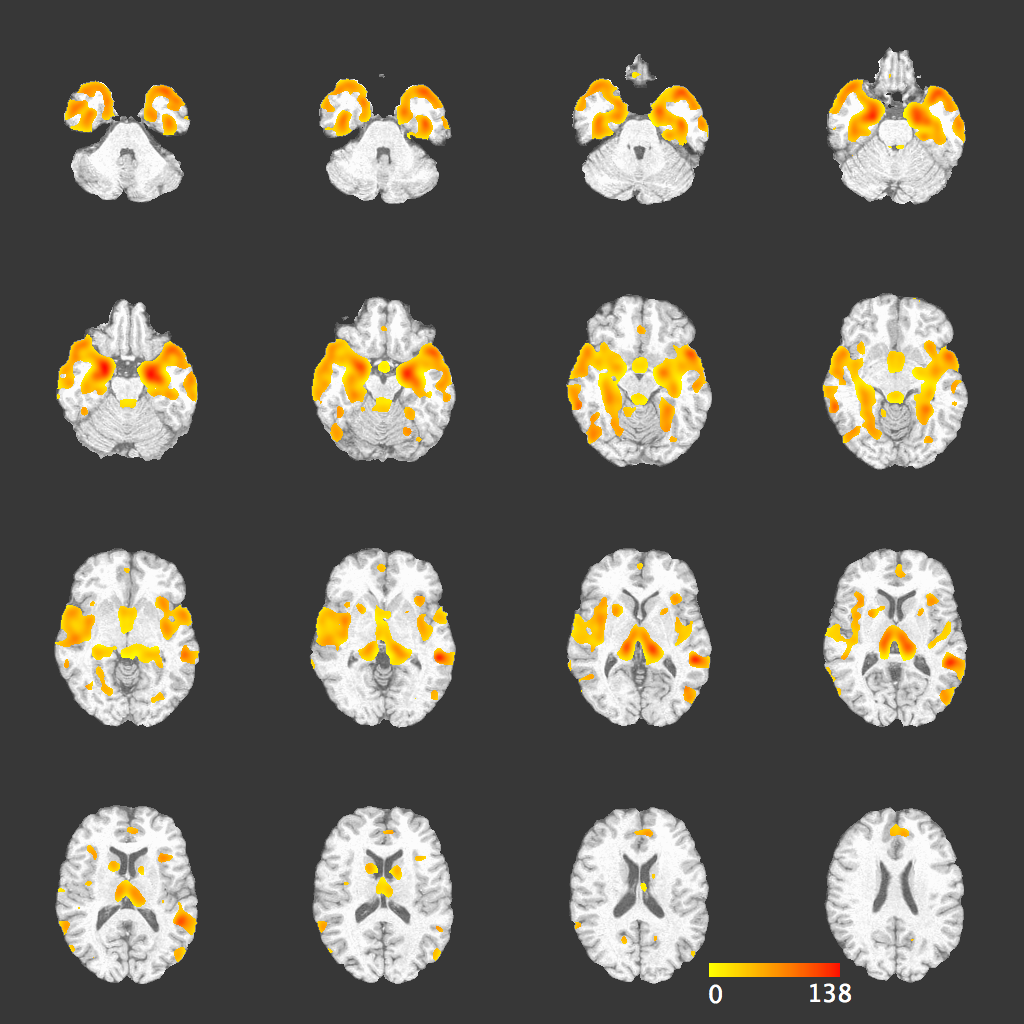


Supplementary Figure 2 Regions of significant group differences in AD related gray matter atrophy between high and low SPARE-AD individuals for subjects older than 65 years old For clarity, only that survived FDR correction at the q<0.001 level are shown, i.e. these are extremely conservative maps.

**Supplementary Results 1**

The association between SPARE-AD and the AD polygenic risk score

The Pearson correlation coefficient between SPARE-AD index and the AD Polygenic risk score was r=0.044 (P=0.069) in the whole age range sample with both imaging and genotyping (n=1,689) and r=0.124 (P=0.016) for subjects’ ≥ 65 years (n=372).
